# Light-Induced Oxidative Stress, *N*-Formylkynurenine, and Oxygenic Photosynthesis

**DOI:** 10.1371/journal.pone.0042220

**Published:** 2012-07-31

**Authors:** Tina M. Dreaden Kasson, Sascha Rexroth, Bridgette A. Barry

**Affiliations:** 1 School of Chemistry and Biochemistry and the Petit Institute for Bioengineering and Bioscience, Georgia Institute of Technology, Atlanta, Georgia, United States of America; 2 Department of Biology, Ruhr-Universität, Bochum, Germany; University of Hyderabad, India

## Abstract

Light stress in plants results in damage to the water oxidizing reaction center, photosystem II (PSII). Redox signaling, through oxidative modification of amino acid side chains, has been proposed to participate in this process, but the oxidative signals have not yet been identified. Previously, we described an oxidative modification, *N*-formylkynurenine (NFK), of W365 in the CP43 subunit. The yield of this modification increases under light stress conditions, in parallel with the decrease in oxygen evolving activity. In this work, we show that this modification, NFK365-CP43, is present in thylakoid membranes and may be formed by reactive oxygen species produced at the Mn_4_CaO_5_ cluster in the oxygen-evolving complex. NFK accumulation correlates with the extent of photoinhibition in PSII and thylakoid membranes. A modest increase in ionic strength inhibits NFK365-CP43 formation, and leads to accumulation of a new, light-induced NFK modification (NFK317) in the D1 polypeptide. Western analysis shows that D1 degradation and oligomerization occur under both sets of conditions. The NFK modifications in CP43 and D1 are found 17 and 14 Angstrom from the Mn_4_CaO_5_ cluster, respectively. Based on these results, we propose that NFK is an oxidative modification that signals for damage and repair in PSII. The data suggest a two pathway model for light stress responses. These pathways involve differential, specific, oxidative modification of the CP43 or D1 polypeptides.

## Introduction

In plants, algae and cyanobacteria, Photosystem II (PSII) catalyzes the photo-oxidation of water to O_2_ and protons [1]. The electrons derived from water are transferred sequentially to two quinone molecules, Q_A_ and Q_B_, on the acceptor side of the reaction center [2]. The cyanobacterial PSII structure was solved to 1.9 Å resolution [2]–[7]. The membrane-spanning D1 and D2 proteins form the core of the reaction center. These proteins bind the catalytic oxygen evolving complex (OEC), which is a Mn_4_CaO_5_ cluster, chlorophyll (chl), pheophytin, and the plastoquinones, Q_A_ and Q_B_, [2]. The CP43 and CP47 proteins are also found in the core of PSII (reviewed in [8]). CP43 and CP47 span the membrane in the PSII complex, and these subunits contain flexible, hydrophilic loops that protrude into the lumen. Substitutions of amino acids in these loops have demonstrated their importance for complex assembly and protection from photoinhibition [8]. Calcium and chloride cofactors are essential for optimal activity under native conditions [9].

Light stress causes protein damage and suboptimal photosynthetic rates in PSII [10], [11]. A decrease in steady state oxygen evolution, as well as accelerated D1 turnover, is the result. Recovery from photoinhibition involves PSII disassembly, proteolysis of damaged D1, and *de novo* synthesis of a new D1 protein. Re-insertion of a new D1 subunit into the partially disassembled PSII complex and reassembly completes the repair cycle [12]. The signaling pathways for complex disassembly and D1 degradation remain unknown. However, post-translational oxidations of amino acids have been proposed to play signaling roles in this process [12].

**Figure 1:**
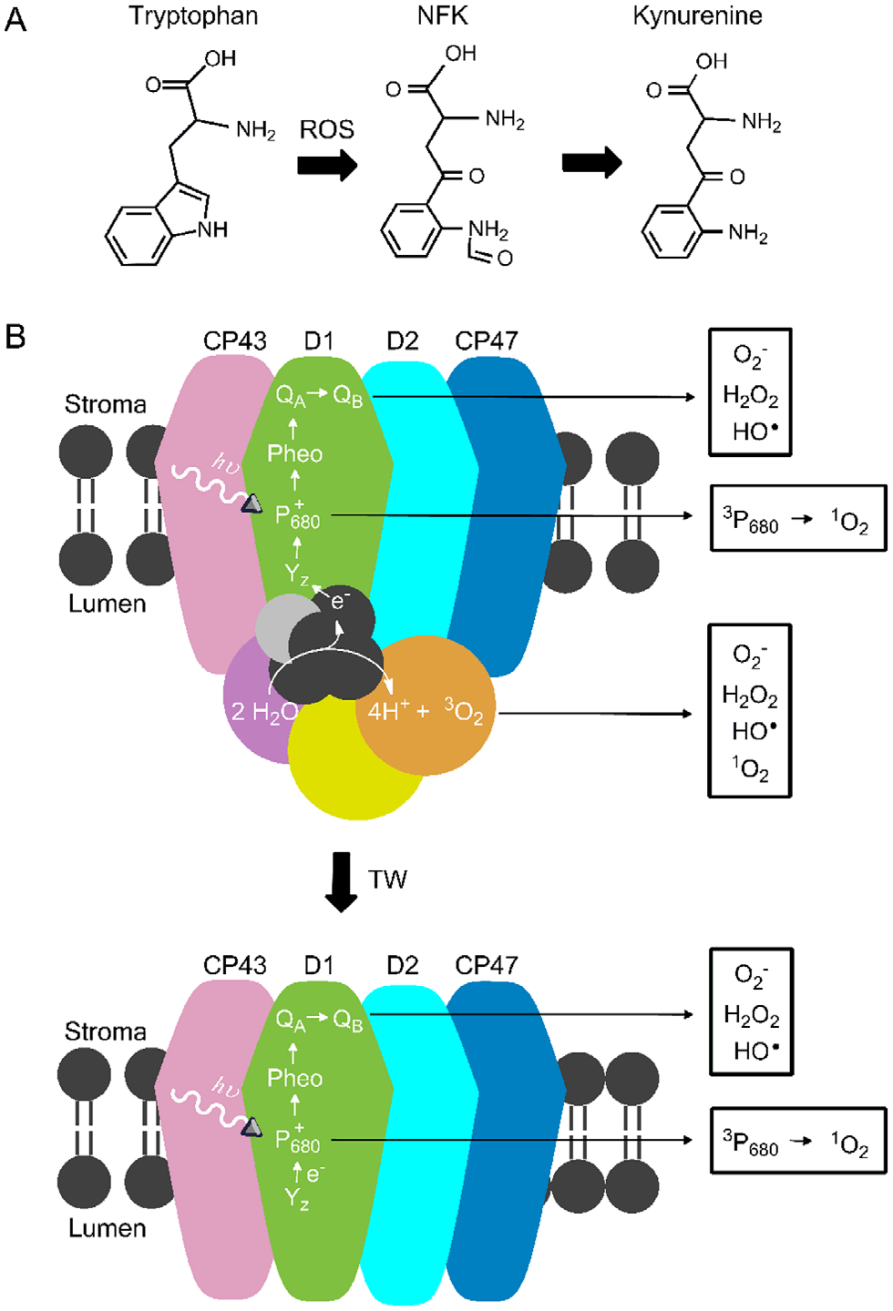
Structures of tryptophan, NFK, kynurenine, and PSII. (A) shows the chemical structures of NFK (+32 *m/z*) and kynurenine (+4 *m/z*). (B) shows models of PSII (top) and TW (bottom) PSII. Tris-washing removes the extrinsic subunits and OEC, or Mn_4_CaO_5_ cluster. The core subunits (CP43, D1, D2 and CP47) and electron transfer cofactors (tyrosine z (Y_z_), P_680_, pheophytin (Pheo), plastoquinone A (Q_A_), and plastoquinone B (Q_B_)) are labeled. The water-splitting reaction at the OEC is shown. The ROS species generated in PSII and TW PSII during photoinhibition are indicated. The subunit colors are the same as in Figure 8. CP43 (pink); D1 (green); D2 (light blue); CP47 (dark blue); extrinsic subunits (violet, yellow, and orange).

Post-translational oxidation of Trp to form *N*-formylkynurenine (NFK) (Figure 1A) plays a role in oxidative stress responses in some proteins (see for example [13]). NFK has been identified in mitochondrial ATP synthase [14], spinach LHCII [15], milk proteins [16], skeletal muscle proteins [17], apolipoprotein B-100 [13], and *Methylococcus capsulatus*-secreted MopE protein [18].

Recently, we described a light-induced modification, NFK (Figure 1A), resulting from the oxidative, post-translational modification (PTM) of W365 in the CP43 subunit [19]. A ∼two fold increase in the yield of NFK365-CP43 was observed following high light illumination [19]. A concomitant two fold decrease in oxygen evolution was detected under the same conditions [19]. This result suggests a role for NFK and oxidative stress in plant photoinhibition.

NFK results from the reaction of the Trp side chain with several types of ROS, including singlet oxygen (^1^O_2_) [15], [20], ozone (O_3_) [21], and hydroxyl radicals (HO^•^) [22], [23]. NFK can also result from a metal-catalyzed radical mechanism, followed by reaction with O_2_ [13]. ROS is produced in PSII, either by recombination reactions producing triplet chlorophyll (^3^chl) or by reactions at the Mn_4_CaO_5_ cluster (Figure 1B). These are referred to as acceptor side (^3^chl) and donor side (Mn_4_CaO_5_) reactions.

In the acceptor side ROS mechanism, double reduction of Q_A_ results in charge recombination and formation of the excited state ^3^chl [24]. Energy transfer from ^3^chl to ground state ^3^O_2_ results in ^1^O_2_ [24]. O_2_ reduction to O_2_^•−^may also occur under light stress conditions [25]. Dismutation to H_2_O_2_, followed by a single electron reduction, may produce HO^•^ [25]. In the donor side ROS mechanism (Figure 1B), release of the extrinsic proteins and OEC during light stress has been reported to stimulate H_2_O_2_ production [26]. The one electron oxidation and reduction of H_2_O_2_ was proposed to produce O_2_^•−^and HO^•^, respectively [25].

In this work, we identify a specific, new oxidative modification of tryptophan in the D1 subunit, which is induced by light-stress. We provide evidence that *N*-formylkynurenine modifications in PSII are generated by ROS, which may be derived from the Mn_4_CaO_5_ cluster. To explain our results, we propose a two-pathway model, in which NFK functions as a signal for D1 protein turnover, a key step in repair under high light stress.

## Results

### Photoinhibition in PSII and Thylakoid Membranes (TMs)

**Figure 2:**
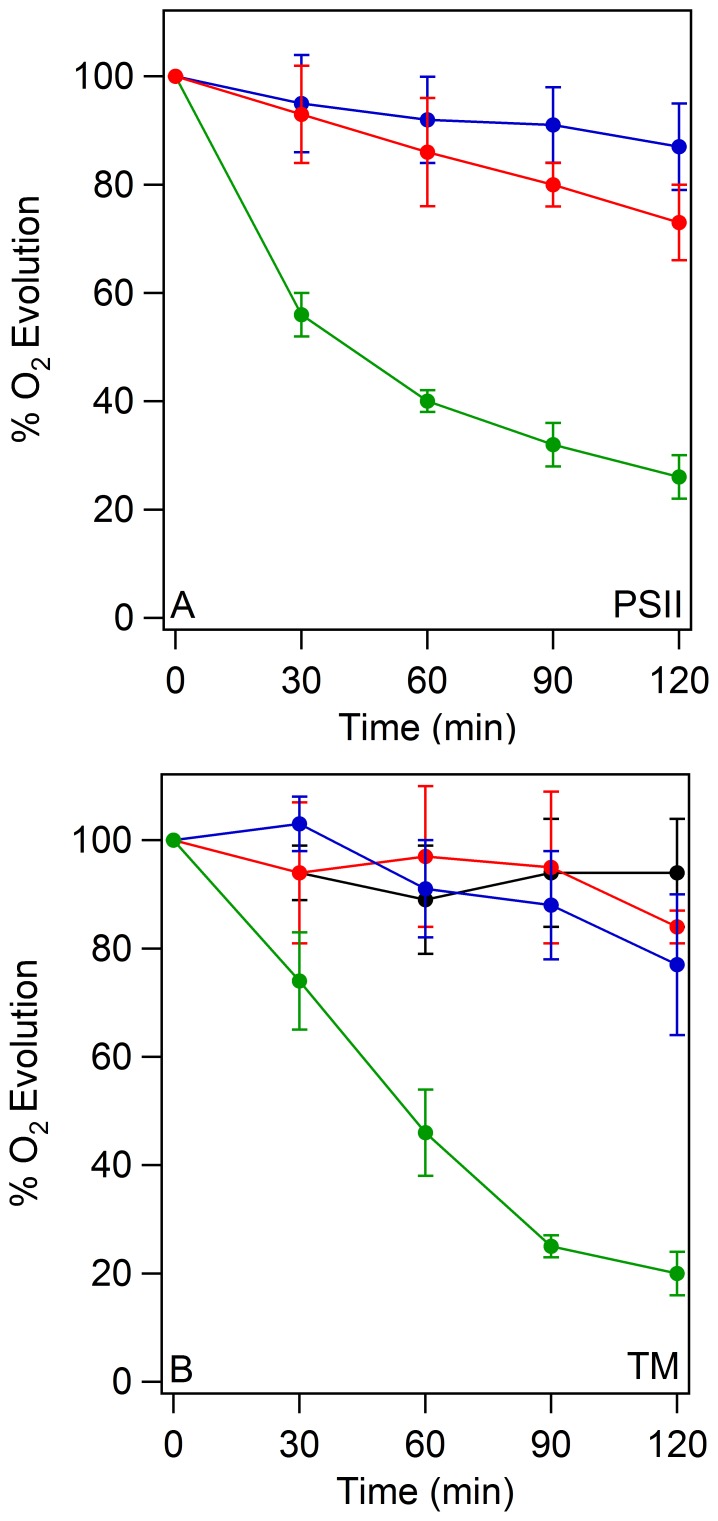
Steady state rates of oxygen evolution of PSII membranes (A) and TM (B) during high light illumination and in the dark. In A, PSII membranes were kept in the dark at 25°C for two hours (blue). PSII membranes were exposed to a white light intensity of 500 (red) and 7,000 (green) µmol photons m^−2^ s^−1^ for two hours at 25°C. In B, TM were kept in the dark (black and red) or exposed to a white light intensity of 7,000 µmol photons m^−2^ s^−1^ at chlorophyll concentrations of 1.0 (blue) or 0.1 mg/ml (green). Oxygen evolution was assayed every 30 minutes and normalized to time zero. The data shown are an average of three to six experiments. The error bars are plus and minus one standard deviation. See Materials and Methods, Photoinhibition, for experimental conditions.

A light intensity of 7,000 µmol photons m^−2^ s^−1^ was employed in these studies. This value is typical of conditions used in previous studies of plant light stress (4,000–7,000 µmol photons m^−2^ s^−1^) [27]–[29]. To evaluate the degree of photoinhibition under these conditions, the steady state rate of oxygen evolution was monitored as a function of illumination time. High light illumination of PSII membranes was conducted at a chlorophyll concentration of 1 mg/mL, pH 6.0, and 25°. Compared to the dark control (Figure 2A, blue), illumination induced a 3.4±0.4 fold decrease in oxygen evolution rate in PSII membranes (Figure 2A, blue and green). This agrees with our previous report of a 2.4±0.5 fold decrease under these conditions [19]. As expected, a lower light intensity of 500 µmol photons m^−2^ s^−1^ did not significantly decrease oxygen evolution rates (Figure 2A, blue and red).

At the same light intensity, thylakoid membrane (TM) samples were not significantly inhibited at a chlorophyll concentration of 1 mg/mL (Figure 2B, black and blue). However, illumination at 0.1 mg/mL chlorophyll induced a 5.4±0.5 decrease in the steady state oxygen evolution rate (Figure 2B, red and green).

### Purification and MS/MS of NFK-containing Peptides in CP43

**Figure 3:**
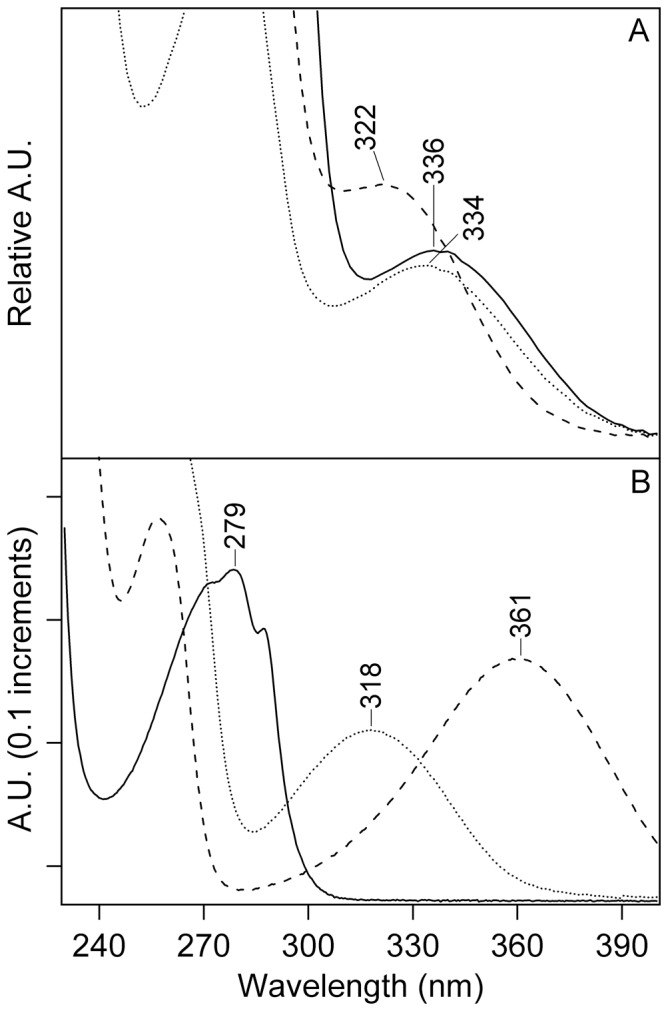
Optical absorption of NFK-containing PSII peptides (A) and the model compounds (B), tryptophan, NFK, and kynurenine. (A) shows absorption spectra of NFK-containing peptide fractions A–C. See Table S1, for average retention times from 350 nm chromatograms. Fraction A is displayed as a solid line, fraction B as a dashed line, and fraction C as a dotted line. In (B), absorption spectra of 40 µM tryptophan (solid line), 40 µM NFK (dotted line), and 40 µM kynurenine (dashed line) are shown in water. Absorption spectra in A were derived from the HPLC chromatogram and are on an arbitrary y-scale (see Materials and Methods). The spectra in B were measured on a Hitachi spectrophotometer (see Materials and Methods).

**Figure 4:**
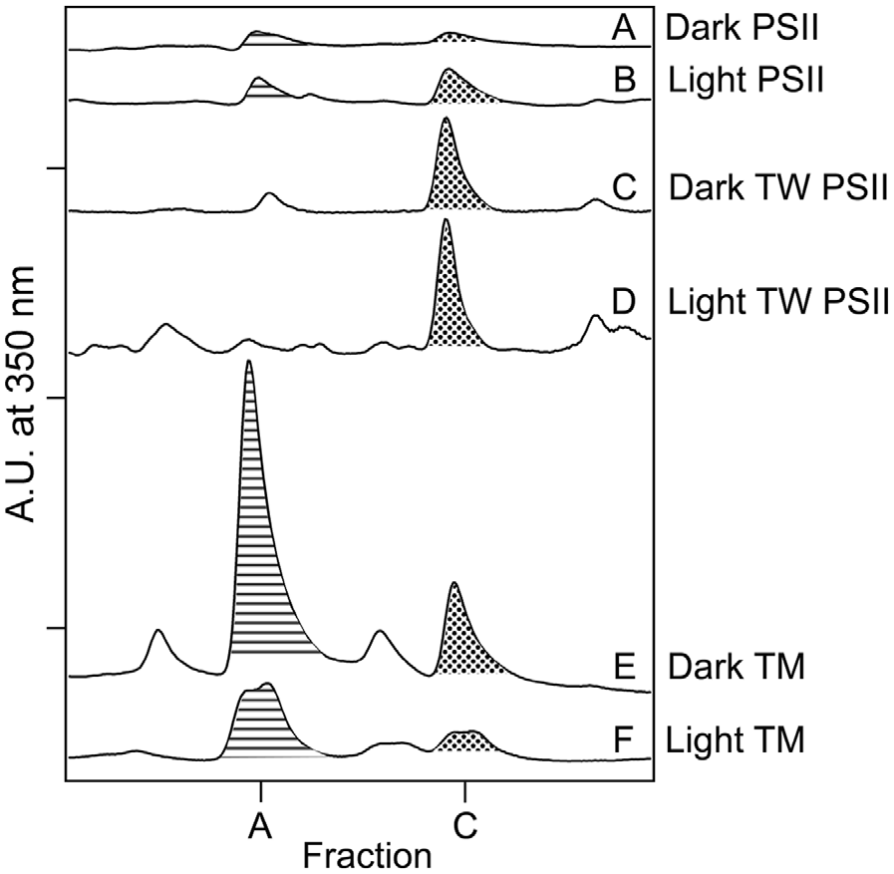
Representative 350 nm HPLC chromatograms of tryptic peptides derived from oxygen-evolving PSII (A–B), OEC-removed (TW) PSII (C–D), and TM (E–F). In (A), (C), and (E), samples were incubated in the dark at room temperature for two hours (control). In (B), (D), and (F), samples were illuminated with ∼7,000 µmol photons m^−2^ s^−1^ of white light for two hours at 25°C. Fraction A is filled with horizontal stripes, and fraction C is filled with dots. The chromatograms are displaced on the y-axis for presentation purposes. The tick increments are 0.085 A.U. See Table S1 for average retention times and summary of light-induced changes. Fraction C corresponds to fraction 1 in ref [19].

NFK has a unique absorption at 318 nm (Figure 3B, dotted line), when compared to tryptophan (Figure 3B, solid line), kynurenine (Figure 3B, dashed line), or other modifications of the indole ring [19]. This unique absorption spectrum allows the identification and purification of NFK-containing tryptic peptides by HPLC (Figure 4). The HPLC chromatogram was monitored at 350 nm during purification of NFK-modified peptides to avoid overlap with the strongly absorbing 280 nm peak. In oxygen-evolving PSII, two different NFK-containing peptides, peptides A and C, were identified (Figure 4A and 4B). Typical absorption spectra, derived from the HPLC chromatograms, are shown in Figure 3A.

Using MS/MS (Table 1 and Figure S1), fraction C (retention time ∼28 min.) was identified as NFK-365 in CP43 (^363^AP(W*)LEPLRGPNGLDLSR^379^), confirming our earlier result [19]. Fraction A contained the same NFK-W365 CP43 modification, but the peptide was shorter, ^363^AP(W*)LEPLR^370^ (Table 1). Only one NFK peptide was detected in Fractions A and C (Table S2). Representative MS/MS data are shown in the Supporting Information (Figure S1).

**Table 1:** MS/MS analysis of NFK modifications in fractions A–C.

Fraction	PSII subunit	Sequence	Modification	MH^+^ (Da)	XCorr
A	CP43	^363^AP(W*)LEPLR^370^	NFK (+32 *m/z*)	1013.5407	2.80
B	D1	^313^VINT(W*)ADIINR^323^	NFK (+32 *m/z*)	1346.7029	3.46
C	CP43	^363^AP(W*)LEPLRGPNGLDLSR^379^	NFK (+32 *m/z*)	1923.0086	2.01

### CP43 NFK in Photoinhibition

**Figure 5:**
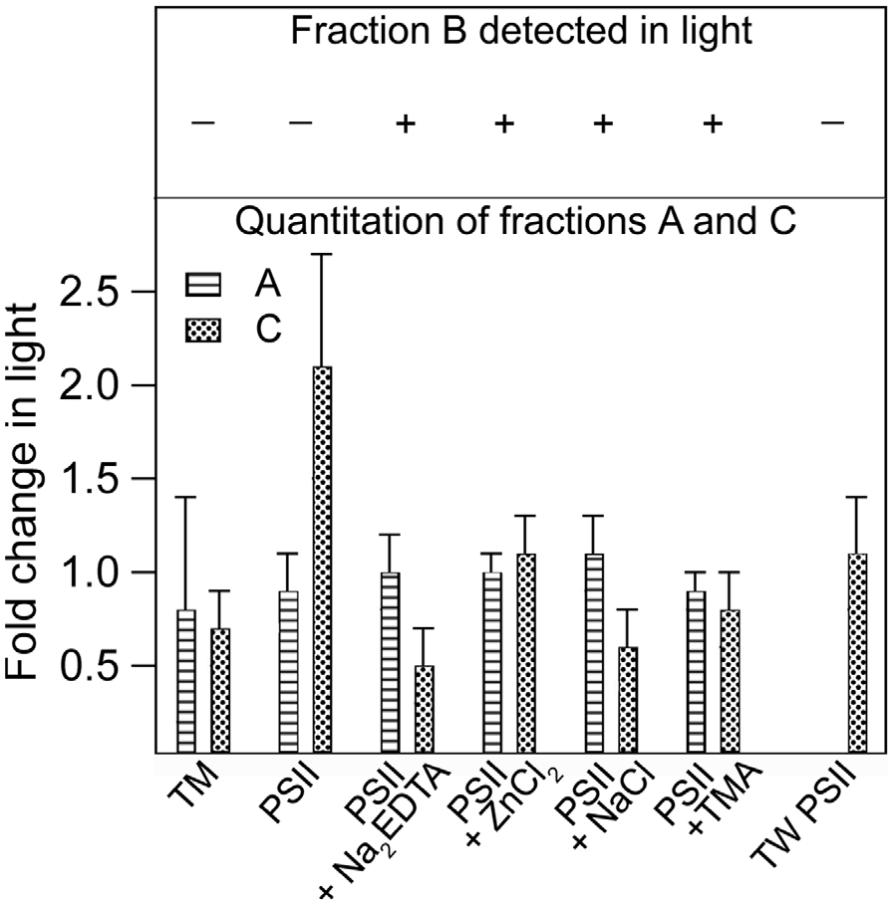
Fraction B detection (top) and yield change in fractions A and C (bottom) following high light illumination. The yields were calculated from the average of three-six different experiments. Peaks in the 350 nm chromatogram were integrated, and the area was divided by the area of the 220 nm chromatogram to correct for changes in yield of tryptic peptides. The error bars are one standard deviation. See Supporting Information (Table S1) for summary of fold changes and average retention times.

To calculate the yield of NFK, the 350 nm peak was integrated, and the value was normalized to the total 220 nm absorption. This corrects for the yield of tryptic peptides [19]. These data are presented in the bar graph shown in Figure 5. As shown, formation of NFK-W365 in fraction C is light induced in oxygen-evolving PSII. The yield increases by 2.1±0.6 (Figure 5). This increase parallels the 3.4±0.4 fold decrease observed in the steady state oxygen evolution rate (Figure 2A, blue and green). However, fraction A (Figure 4A and 4B) does not show a significant light-induced increase (0.9±0.2, Figure 5 and Table S1).

In TW PSII, the Mn_4_CaO_5_ cluster and extrinsic subunits are removed [30]. Under these conditions (Figure 1B), without active oxygen evolution, no significant light induced increase is observed in fraction C (Figures 4C and 4D, Figure 5). Fraction A is not observed in TW PSII.

### NFK365-CP43 is Observed in TM, but does not Show a Light-induced Increase

In TM samples, fractions A and C are observed in the dark and the light (Figure 4E and 4F). Field grown spinach leaves, exposed to unregulated growth conditions, were used for the TM isolation. Thus, NFK modifications may be present in the dark, due to the previous handling of the market spinach. The observation of NFK in TM demonstrates that the modification is not induced by detergent treatment. There is no significant, light-induced increase in these fractions (Figure 5 and Table S1). This parallels the results of the oxygen evolution assays conducted under the same conditions (1 mg/mL chlorophyll) in Figure 2B (black and blue), which showed that the TM preparation was resistant to photoinhibition.

### Photoinhibition at Increased Ionic Strength Results in a New NFK-containing Peptide, Peptide B

**Figure 6:**
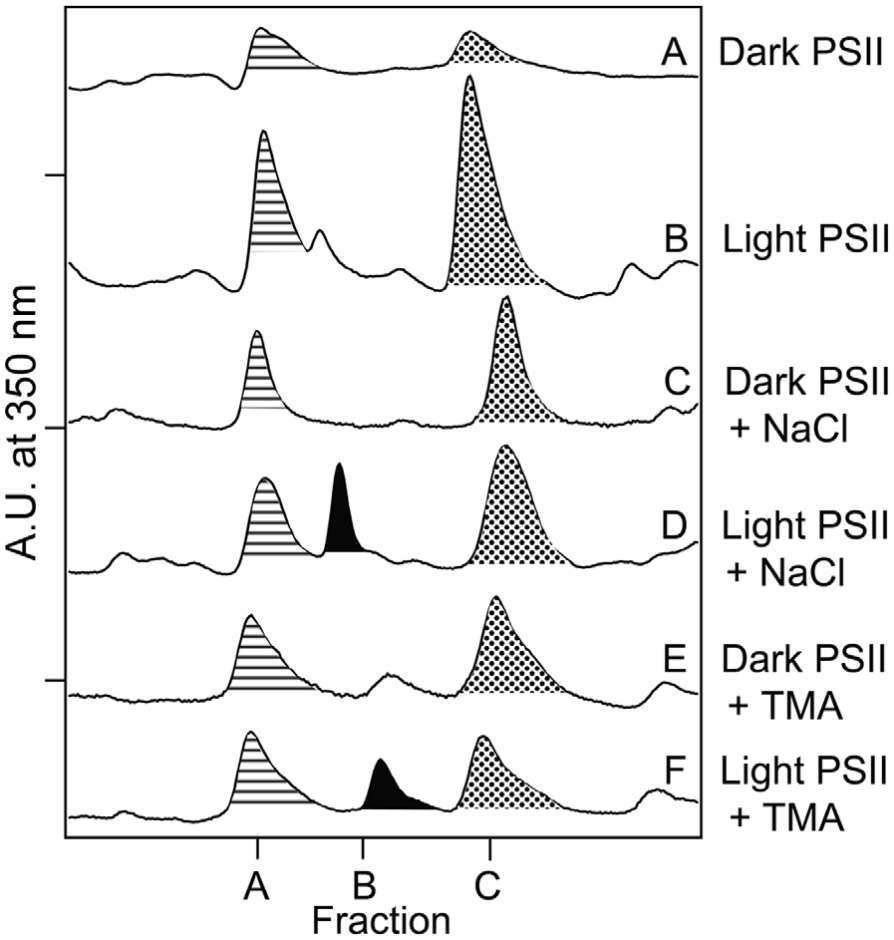
Representative 350 nm HPLC chromatograms of oxygen-evolving PSII with and without 2 mM NaCl or TMA. In (A), (C), and (E), samples were incubated in the dark at room temperature for two hours (controls). In (B), (D), and (F), samples were illuminated with ∼7,000 µmol photons m^−2^ s^−1^ of white light for two hours at 25°C. In (C) and (D), 2 mM NaCl was added just prior to the dark or light incubation. In (E) and (F), 2 mM TMA was added just prior to the dark or light incubation. Fraction A is filled with horizontal stripes, fraction B has solid fill, and fraction C is filled with dots. The chromatograms are displaced on the y-axis for presentation purposes. The tick increments are 0.020 A.U. See Supporting Information for average retention times and summary of light-induced changes (Table S1). Fraction C corresponds to fraction 1 in ref [19].

The photoinhibition experiment was conducted on PSII membranes in SMN buffer to which 2 mM NaCl was added. Compared to PSII in SMN buffer (Figures 6A and 6B), a new peptide, peptide B, was observed in the light (Figures 6C and 6D). Fraction B was not observed in the dark (Figure 6C). Under these conditions there was no significant increase in the yield of fractions A or C (Figure 5). MS/MS identified peptide B as ^313^VINT(W*)ADIINR^323^ in D1 (Tables 1 and S2, Figure S1). Only one NFK peptide was detected in this fraction (Table S2).

To test if the observation of peptide B depended on the identity of the cation or anion, the experiment was conducted in the presence of ZnCl_2_ (0.15 mM) and Na_2_EDTA (1 mM). Peptide B was observed under both sets of conditions (Figure 5). This result is not consistent with a role for a specific mono- or divalent ion.

To rule out the possibility of a non-specific cation-binding site as inducing the fraction B modification, the effect of 2 mM tetra-methyl ammonium chloride (TMA) was assessed. TMA has a nearly three-fold larger ionic radius (2.9 Å) [31] when compared to Na^+^ (1.0 Å) [31] or Zn^2+^ (0.74 Å) [32]. TMA could not replace Ca^2+^ (ionic radius  = 0.99 Å) [33] in PSII [34] or β-1,4-glucanase [35]. In our experiments, we found that 2 mM TMA also induced fraction B in the light (Figure 5F).

We conclude that the small ionic strength increase underlies the observation of fraction B. The ionic strength of the SMN buffer, prior to the addition of salts, is calculated to be 34.9 mM. The ionic strength increased to 36.9 mM (2 mM NaCl or TMA), 35.4 mM (0.15 mM ZnCl_2_), and 36.4 mM (1 mM Na_2_EDTA) when peptide B was observed in the light.

Approximately 7% of TW PSII reaction centers were reported to contain CP43 NFK-365 [19]. Assuming the same extinction coefficients (3750 M^−1^ cm^−1^ at 321 nm [36], the yield of D1 NFK-317 (fraction B) can be estimated. Comparison of HPLC peak intensities indicates that approximately 1% of the PSII centers contain D1 NFK-317 after photoinhibition.

### Photoinhibition of PSII Membranes is Associated with D1 Oligomerization and Proteolysis

**Figure 7:**
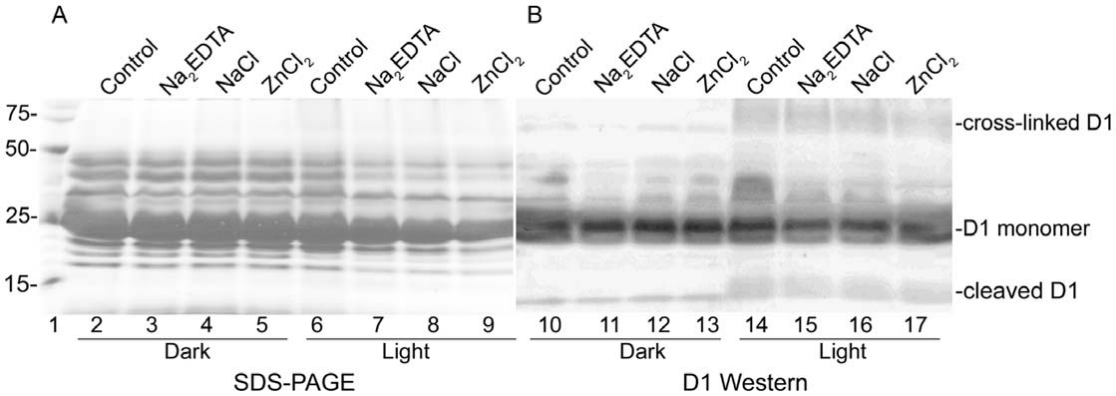
SDS-PAGE (A) and Western blot (B) using an antibody specific for the C-terminus of the D1 protein (Agrisera) (B). Control PSII membranes were maintained in the dark (lanes 2–5; lanes 10–13) or exposed to high light (∼7,000 µmol photon m^−2^ s^−1^) for two hours at 25°C (lanes 6–9; lanes 14–17). Samples were either untreated (lanes 2, 6, 10, 14) or treated with 1 mM Na_2_EDTA (lanes 3, 7, 11 and 15), 2 mM NaCl (lanes 4, 8, 12, and 16), or 0.15 mM ZnCl_2_ (lanes 5, 9, 13, and 17). Lane 1 displays the molecular weight markers. In both dark and light experiments, 24 µg chl was loaded per lane.

Figure 7 shows SDS-PAGE and Western analysis, comparing the reaction of an anti-D1 antibody with PSII membranes. D1 oligomers and proteolytic fragments were observed after illumination. Illumination in the presence of increased NaCl, ZnCl_2_, and Na_2_EDTA gave the same result (Figure 7).

### An Additional Light-induced NFK Modification is Observed in PSII

An additional NFK peptide was detected with a 34 min retention time (Tables S1 and S2). This fraction (D) increased in intensity in the light. More than one NFK peptide was detected in this fraction, with one identified as the D2 polypeptide ^8^FTKDEKDLFDSMDD(W*)LR^24^ and the other identified as the D2 polypeptide ^14^DLFDSMDD(W*)LR^24^ (Tables S1 and S2). In our previous work, which employed HPLC and affinity purification, an NFK modification of a light-harvesting subunit was detected with a similar retention time. Due to the complexity of this fraction, interpretation of the light induced increase in fraction (D) awaits further experimentation.

## Discussion

### Summary

In this work, three NFK containing peptides, originating from the donor side of PSII, are identified. Fraction A, corresponds to ^363^AP(W*)LEPLR^370^ in CP43 and is observed in oxygen-evolving PSII and TM, but not in TW PSII. Fraction A showed no detectable light-induced increase in any sample that we examined. Fraction C, corresponds to ^363^AP(W*)LEPLRGPNGLDLSR^379^ in CP43, and is observed in oxygen evolving PSII, TW PSII, and TM. Fraction C showed a light induced increase in only one sample, oxygen-evolving PSII. Fraction B corresponds to ^313^VINT(W*)ADIINR^323^ in D1. It was observed only in oxygen-evolving PSII, after illumination and under conditions of higher ionic strength.

### Location of the NFK Modifications

**Figure 8:**
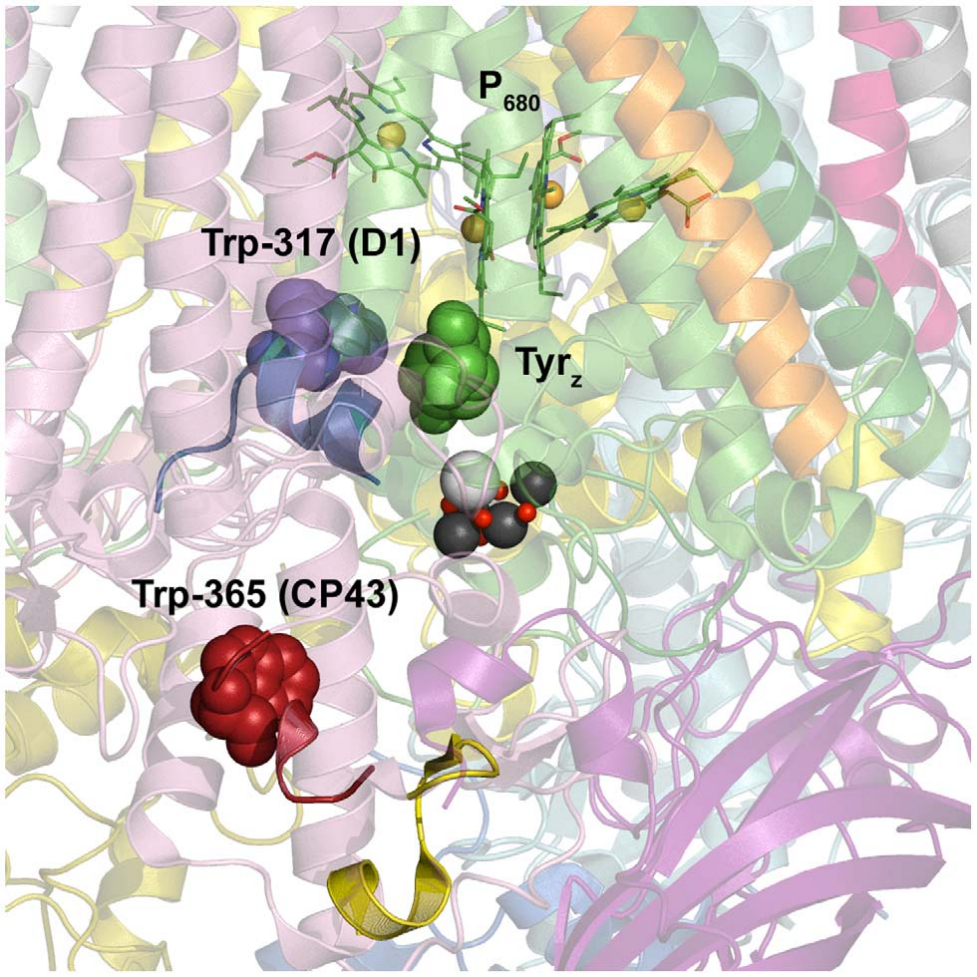
Predicted locations of NFK modifications, NFK365-CP43 and NFK317-D1, in the *T. vulcanus* PSII structure [**2**]. The OEC is shown in black, grey, and red. P_680_ and YZ (green spacefill) are shown above the OEC. The CP43 and D1 backbones are displayed in pink and green, respectively. The side chain of Trp-365 in CP43 is in red spacefill. The side chain of Trp-317 in D1 is in blue spacefill. MS/MS detected tryptic peptides corresponding to fraction A (red and yellow combined), B (blue), and C (red) are highlighted. The image was rendered with the Pymol Molecular Grapics System (www.pymol.org).

Figure 8 shows the position of the NFK modifications in the PSII structure from *T. vulcanus* [2]. NFK317-D1 is located ∼24 Å away from NFK365-CP43. NFK 365-CP43 is 17 Å from the Mn_4_CaO_5_ cluster; NFK 317-D1 is 14 Å from the cluster. Figure 8 also shows the position of NFK365-CP43 and NFK317-D1 relative to P_680_ and YZ. YZ is an electron transfer intermediate during the water oxidizing reaction (Figure 1B) [37]. YZ is oxidized by the primary chlorophyll donor, P_680_, and in its radical form, YZ is a strong oxidant [38]. However, NFK365-CP43 is 19 Å from YZ and 30 Å from P_680_. NFK317-D1 is 27 Å from YZ and 19 Å from P_680_. Thus, a YZ or P_680_ radical-based mechanism for the formation of the NFK modifications seems unlikely.

Sequence conservation across photosynthetic organisms supports an important evolutionary role for both NFK-modified tryptophans. Although these core subunits are consistent in plants and cyanobacteria, some distinct structural distinctions remain. These differences mainly lay in the extrinsic [39] and light harvesting antennae [40] polypeptides. Thus, the location and role of NFK in cyanobacteria remains to be determined.

### ROS and Specificity of NFK Modifications in PSII and Other Proteins

In our experiments, we attribute the formation of NFK to the reaction of the Trp side chain with ROS [41]. Studies in mitochondrial proteins have concluded that the NFK modification is a ROS-targeted mechanism [14], [42], [43]. In mitochondrial ATP synthase, the NFK modification was channeled to a single Trp residue (Trp-503) [14]. In the mitochondrial aconitase-2 protein, site-specific oxidation of Trp-373 was also observed [42]. The specificity of the post-translational NFK modification in PSII and other proteins suggests a selective physiological role for the modification.

We demonstrate here that removal of the Mn_4_CaO_5_ cluster and extrinsic subunits prevents light-induced accumulation of NFK in the CP43 subunit. Previously, EPR spin trapping experiments have suggested that photoinhibited oxygen evolving PSII produces both ^1^O_2_ and HO^•^ [44]. However, only HO^•^ was detected in Mn-depleted PSII [44]. Coupled with our data, this previous result is supportive of the conclusion that ^1^O_2_ [15], and not HO^•^ [22], reacts with Trp to form NFK. Our results suggest a Mn_4_CaO_5_ origin for the reactive oxygen species, which oxidizes the Trp side chain. However, we cannot rule out the possibility that extrinsic subunit removal or acceptor side alterations are contributing factors.

### NFK in D1 Turnover and Photoinhibition

Photoinhibition is known to induce D1 protein damage and a high rate of D1 turnover [10], [12], [45]. Previous studies have identified intermolecular cross-links of D1 with D2, cytochrome b_559_, and CP43 [46], [47]. In intact leaves and chloroplasts, D1 damage and turnover also occurred by D1 fragmentation and cross-linking [46]. These cross-links were proposed to participate in pathways for complete degradation of damaged D1 *in vivo* [46], [47]. NFK can bind covalently to primary amine-containing side chains [19], such as arginine and lysine, and may participate in this proteolysis pathway. The Western blot analysis of PSII membranes, presented here, confirmed that D1 cleavage and oligomerization occurred when NFK accumulated either in CP43 or in D1. The 3.4±0.4 decrease in oxygen evolution rate of PSII membranes under the same conditions is further support for a correlation of photoinhibitory effects with NFK formation.

Reversible, light induced structural changes in the degree of spinach TM stacking (grana) have been observed by electron microscopy [48]. These dynamic alterations in structural organization may be involved in protection from light stress [49] and would not occur in isolated PSII membrane fractions. In future work, we will explore the impact of these topological changes. In these experiments, we compared TM with PSII for two reasons. First, we wished to examine the possibility that the NFK modification is induced by detergent treatment. Observation of the NFK modification in TM in the dark eliminates this possibility. The residual level of oxidative modification in the dark may be due to the use of market, field grown spinach, which is transported and harvested under uncontrolled conditions. Second, TM samples do not photoinhibit at the high chl concentrations necessary for the HPLC assay. Therefore, TM preparations provide an important negative control for the PSII experiments. We report that illumination of TM did not accumulate NFK, supporting the conclusion that the increase in NFK yield is caused by light stress. We attribute the resistance to photoinhibition in the TM to a shading effect [50] **,** because illumination of TM with the same light intensity at a lower chlorophyll concentration (0.1 mg/mL) significantly decreased activity.

### Effects of Ionic Strength on Oxidative Modifications

In this work, we found the interesting result that small ionic strength increases had a dramatic effect on the pattern of NFK modifications. However, this change did not alter the degradation pattern of D1 as assessed with a C-terminal antibody. Although the D1 degradation pattern was not changed, the alternative D1-NFK modification to Trp-317 was induced by increasing ionic strength. The ionic strength effect may cause conformational changes in the extrinsic loops. Lowering of the thylakoid lumen pH during excess light involves protein conformational changes that may be necessary in non-photochemical quenching [51].

Concomitant Mg^2+^ efflux into the stroma occurs during the transition from dark to light conditions [52], [53]. Thus, ionic strength induced events are essential in regulatory pathways in TM and PSII. Further evidence for loop dynamics is provided by the inefficient tryptic cleavage of CP43 in TW PSII, noted here. For example, both the ^363^AP(W*)LEPLR^370^ peptide (fraction A) and the ^363^AP(W*)LEPLRGPNGLDLSR^379^ (fraction C) peptide were observed in intact PSII and TM. However, in TW PSII, the shorter CP43 peptide ^363^AP(W*)LEPLR^370^ was not detected. These results can be attributed to different conformations of the CP43 loop region. These conformational changes may be important in control of photoinhibitory responses in the chloroplast, where changes in the proton motive force can occur during illumination.

### NFK in PSII Signaling and Repair

The signaling pathways for induction and control of D1 turnover are not known. Oxidative PTMs of aromatic amino acids have been proposed to participate in signaling. The NFK modifications identified here may function as these signals. We showed previously that substitutions at Trp-365 (Trp-352 in *Synechocystis* 6803) did not affect the steady-state rate of oxygen evolution under normal light-saturated conditions [54]. This result indicated that mutations at Trp-365 do not alter the structure of PSII or change the overall rates of electron transfer. However, the mutants displayed an increased rate of photoinhibition at higher light intensities (5,000 µmol photons m^−2^ s^−1^) [55]. Thus, the inability to form NFK in the mutants resulted in reduced repair during high light stress. Because the light-induced increases in NFK in CP43 and D1 appear to be mutually exclusive, we propose that these modifications occur on two different damage/repair pathways. Inhibition of the CP43 pathway promotes the D1 oxidative pathway.

The primary proposed protease involved in D1 turnover, FtsH [56], [57], has been proposed to recognize partially unfolded proteins [58]. Oxidation of Trp to NFK may promote partial protein unfolding required for signal recognition by the protease [56]. Replacement of NFK with unmodified Trp requires *de novo* protein synthesis [59]. Multiple NFK modifications may be required for continuous D1 turnover. Interestingly, an increase in CP43 degradation and cross-linking was observed during photoinhibition and donor side inactivation [29].

### Conclusions

Our data provide evidence for specific oxidative modifications of PSII subunits. These PTMs are induced by high light stress and are under differential control of ionic strength. We propose that NFK plays a role in signaling for repair during D1 turnover. In a two-pathway signaling model for repair, inhibition of one NFK signaling pathway (the “CP43” pathway) stimulates repair by the alternative pathway (the “D1” pathway). These results provide new insight into redox signaling in oxygenic photosynthesis.

## Materials and Methods

### Thylakoid, PSII, and TW PSII Membrane Preparations

Spinach PSII membranes were isolated as described [60] with modifications [61]. Thylakoid membrane (TM) isolation was conducted as described in [60], with a single centrifugation and wash after the initial grinding step. The TM wash buffer was 20 mM 2-(*N*-morpholino)-ethanesulfonic acid (MES)-NaOH (pH 6.0), 150 mM NaCl, 4 mM MgCl_2_•H_2_O. The final resuspension was in 50 mM MES-NaOH (pH 6.0), 400 mM sucrose, 15 mM NaCl (SMN buffer). Chlorophyll [62] and oxygen assays were conducted as described [54]. Oxygen evolution experiments were conducted with red-filtered light from a Dolan-Jenner (Boxborough, MA) Fiber-Lite illuminator at 25°C in SMN buffer with 1 mM K_3_Fe(CN)_6_ and recrystallized 1 mM 2,6-dichlorobenzoquinone (DCBQ). Activity rates of PSII membranes and TM were ≥600 and ≥130 µmol O_2_ mg chl^−1^ h^−1^, respectively.

The 18- and 24-kDa extrinsic subunits were removed from PSII membranes with a 2 M NaCl wash [63]. PsbO and the Mn_4_CaO_5_ cluster were removed with a 800 mM tris(hydroxymethyl)aminomethane (Tris)-NaOH, pH 8.0 wash performed for 45 minutes at room temperature in the light [30]. Tris-washed (TW) PSII membranes were washed three times with a buffer of 400 mM sucrose, 50 mM 4-(2-hydroxyethyl)-1-piperazineethanesulfonic acid (HEPES)-NaOH, (pH 7.5) (SH buffer). TW PSII samples were resuspended in the same buffer at a chlorophyll concentration of 2–4 mg/mL chlorophyll.

### Photoinhibition

Photoinhibition experiments were performed on spinach TM, PSII membranes, and TW PSII membranes [19]. The final resuspension buffers were used during illumination (see Materials and Methods, Thylakoid, PSII, and TW PSII membrane preparations above). All samples were stirred and kept at 25°C with a water bath and dewar, during white light illumination with a Dolan-Jenner (Boxborough, MA) Fiber-Lite illuminator. Illumination of PSII was conducted at a chlorophyll concentration of 1.0 mg/mL. TM were illuminated at 1.0 or 0.1 mg/mL chlorophyll. The light intensity used was ∼7,000 µmol photons m^−2 ^s^−1^ as measured with a Li-Cor (Lincoln, NE) Light Meter (model LI-189 with a ∼8 cm diameter sensor). The illumination was performed for two hours. Controls were kept in the dark at room temperature (∼25°C). Samples were either un-treated or treated with 2 mM NaCl (Fisher Scientific, Fairlawn, NJ), 0.15 mM ZnCl_2_ (BDH VWR, Radnor, PA), 1 mM disodium-ethylenediaminetetraacetic acid (Na_2_EDTA) (JT Baker, Austin, TX), or 2 mM tetra-methylammonium chloride (TMA) (Sigma-Aldrich, St. Louis, MO) by addition just prior to the dark or light incubation. The ionic strength of the SMN buffer alone, prior to the addition of salts, was 34.9 mM. The ionic strength increased to 36.9 mM (2 mM NaCl and 2 mM TMA), 35.4 mM (0.15 mM ZnCl_2_), and 36.4 mM (1 mM Na_2_EDTA) with the additional salts. Oxygen evolution was assayed every 30 minutes.

### UV-Visible Spectrophotometry

Optical spectra of model compounds tryptophan, NFK, and kynurenine were recorded at room temperature from 200–750 nm on a Hitachi (U3000) spectrophotometer [19]. The model compounds, 40 µM *L*-tryptophan (Sigma-Aldrich, St. Louis, MO), *L*-kynurenine (Sigma-Aldrich), and NFK [19], [64] were suspended in H_2_O. The NFK-containing peptide optical spectra were derived from the chromatogram through the use of a Beckman (Brea, CA) System Gold® HPLC, equipped with a 125 solvent module, a 168 photodiode array detector (1 cm path length, 2 nm scan interval), and 32 Karat Software, version 7.0.

### Tryptic Peptide Digestion and High Pressure Liquid Chromatography (HPLC) Assay

*In-situ* trypsin (Life Technologies, Carlsbad, CA) digestion of TM, PSII, and TW PSII was conducted as described [19]. HPLC separation, isolation of NFK-containing peptides, and quantitative NFK assay were carried out as previously described [19]. Retention times for Fractions A–D were 25, 26, 28, and 34 minutes and were reproducible to ±0.6 min (Table S1). The amount of the NFK containing peptide was quantitated by integration of the 350 nm peak by the procedure previously described [19]. This area was normalized to the total 220 nm absorption. This normalization corrects for any differences in the yield of tryptic products (Table S1).

### Sodium Dodecyl Sulfate Polyacrylamide Gel Electrophoresis (SDS-PAGE) and D1 Protein Western Blot

SDS-PAGE of PSII membranes was performed as described [65]–[67]. 24 µg of chl were loaded per lane. Following SDS-PAGE, gels were either stained with 0.05% Brilliant Blue R (Coomassie) (Sigma-Aldrich, St. Louis, MO) or used for D1 Western blot analysis. For the Western blot, an unstained gel was blotted onto a 0.45 µm polyvinylidene fluoride (PVDF) membrane by semi-dry transfer as described [68]. A PSII D1 (PsbA) C-terminal antibody (Agrisera, Vannas, Sweden) (1∶10,000 dilution) was used as the primary antibody probe. A secondary anti-chicken-alkaline phosphatase conjugate (Sigma-Aldrich, St. Louis, MO) was the secondary antibody probe (1∶15,000 dilution). A 5-bromo-4-chloro-3-indolyl phosphate/nitro blue tetrazolium (BCIP/NBT) liquid substrate system (Sigma-Aldrich, St. Louis, MO) was used for colorimetric detection.

### Tandem Mass Spectrometry (MS/MS) Peptide Analysis

PSII tryptic peptides were analyzed as described [19]. Representative MS/MS data are shown in Figure S1.

## Supporting Information

Figure S1 **Representative MS/MS spectra of NFK modifications in CP43 (A and C) and D1 (B) proteins.** The peaks in blue represent the *b*-fragments. The peaks in red represent the *y*-fragments. The NFK modified W is indicated in the corresponding sequences. This residue carries the +32 *m*/*z* masss shift, which was unambiguously assigned to Trp-365 in CP43 (A and C) and Trp-317 in D1 (B).
(DOCX)

Table S1 **^#^NOD, not observed in dark; NODL, not observed in dark or light.**
(DOCX)

Table S2 **For the identification of peptides, filter criteria were set to warrant a false discovery rate of less than 1% on the peptide level.** In each of the three independent LC-MS/MS runs of the four fractions, more than 20000 MS/MS spectra were recorded. For fraction A–C, between 3500–5000 spectra were assigned to peptides from 50–80 proteins from *S. oleracea*. In fraction D only, 1200 spectra could be assigned to peptides of about 50 proteins.
(DOCX)

## References

[1] Nelson N, Yocum CF (2006) Structure and function of photosystems I and II. Annu Rev Plant Biol 57: 521–565. 16669773 10.1146/annurev.arplant.57.032905.105350

[2] Umena Y, Kawakami K, Shen J-R, Kamiya N (2011) Crystal structure of oxygen-evolving photosystem II at a resolution of 1.9 Å. Nature 473: 55–60. 21499260 10.1038/nature09913

[3] Ferreira KN, Iverson TM, Maghlaoui K, Barber J, Iwata S (2004) Architecture of the photosynthetic oxygen-evolving center. Science 303: 1831–1838. 14764885 10.1126/science.1093087

[4] Guskov A, Kern J, Gabdulkhakov A, Broser M, Zouni A, et al (2009) Cyanobacterial photosystem II at 2.9 Å resolution and the role of quinones, lipids, channels and chloride. Nat Struct Mol Biol 16: 334–342. 19219048 10.1038/nsmb.1559

[5] Kamiya N, Shen J-R (2003) Crystal structure of oxygen-evolving photosystem II from *Thermosynechococcus vulcanus* at 3.7 Å resolution. Proc Natl Acad Sci U S A 100: 98–103. 12518057 10.1073/pnas.0135651100 PMC140893

[6] Loll B, Kern J, Saenger W, Zouni A, Biesiadka J (2005) Towards complete cofactor arrangement in the 3.0 Å resolution structure of photosystem II. Nature 438: 1040–1044. 16355230 10.1038/nature04224

[7] Zouni A, Witt H-T, Kern J, Fromme P, Krauß N, et al (2001) Crystal structure of photosystem II from *Synechococcus elongatus* at 3.8 Å resolution. Nature 409: 739–743. 11217865 10.1038/35055589

[8] Bricker TM, Frankel LK (2002) The structure and function of CP47 and CP43 in photosystem II. Photosynth Res 72: 131–146. 16228513 10.1023/A:1016128715865

[9] Yocum CF (2008) The calcium and chloride requirements of the O_2_ evolving complex. Coord Chem Rev 252: 296–305.

[10] Tyystjärvi E (2008) Photoinhibition of photosystem II and photodamage of the oxygen evolving manganese cluster. Coord Chem Rev 252: 361–376.

[11] Yamamoto Y, Aminaka R, Yoshioka M, Khatoon M, Komayama K, et al (2008) Quality control of photosystem II: impact of light and heat stresses. Photosynth Res 98: 589–608. 18937045 10.1007/s11120-008-9372-4

[12] Nixon PJ, Michoux F, Yu J, Boehm M, Komenda J (2010) Recent advances in understanding the assembly and repair of photosystem II. Ann Bot 106: 1–16. 20338950 10.1093/aob/mcq059 PMC2889791

[13] Gießauf A, van Wickern B, Simat T, Steinhart H, Esterbauer H (1996) Formation of *N*-formylkynurenine suggests the involvement of apolipoprotein B-100 centered tryptophan radicals in the initiation of LDL lipid peroxidation. FEBS Lett 389: 136–140. 8766816 10.1016/0014-5793(96)00546-7

[14] Rexroth S, Poetsch A, Rögner M, Hamann A, Werner A, et al (2012) Reactive oxygen species target specific tryptophan site in the mitochondrial ATP Synthase. Biochim Biophys Acta 1817: 381–387. 22133636 10.1016/j.bbabio.2011.11.006

[15] Rinalducci S, Campostrini N, Antonioli P, Righetti PG, Roepstorff P, et al (2005) Formation of truncated proteins and high-molecular-mass aggregates upon soft illumination of photosynthetic proteins. J Proteome Res 4: 2327–2337. 16335982 10.1021/pr0502368

[16] Ehrenshaft M, Silva SdO, Perdivara I, Bilski P, Sik RH, et al (2009) Immunological detection of *N*-formylkynurenine in oxidized proteins. Free Radic Biol Med 46: 1260–1266. 19353782 10.1016/j.freeradbiomed.2009.01.020 PMC2891935

[17] Fedorova M, Todorovsky T, Kuleva N, Hoffmann R (2010) Quantitative evaluation of tryptophan oxidation in actin and troponin I from skeletal muscles using a rat model of acute oxidative stress. Proteomics 10: 2692–2700. 20455213 10.1002/pmic.201000147

[18] Helland R, Fjellbirkeland A, Karlsen OA, Ve T, Lillehaug JR, et al (2008) An oxidized tryptophan facilitates copper binding in *Methylococcus capsulatus*-secreted protein MopE. J Biol Chem 283: 13897–13904. 18348978 10.1074/jbc.M800340200

[19] Dreaden TM, Chen J, Rexroth S, Barry BA (2011) *N*-formylkynurenine as a marker of high light stress in photosynthesis. J Biol Chem 286: 22632–22641. 21527632 10.1074/jbc.M110.212928 PMC3121407

[20] Gracanin M, Hawkins CL, Pattison DI, Davies MJ (2009) Singlet-oxygen-mediated amino acid and protein oxidation: formation of tryptophan peroxides and decomposition products. Free Radic Biol Med 47: 92–102. 19375501 10.1016/j.freeradbiomed.2009.04.015

[21] Previero A, Coletti-Previero M-A, Jollès P (1967) Localization of non-essential tryptophan residues for biological activity of lysozyme. J Mol Biol 24: 261–268. 6030026 10.1016/0022-2836(67)90331-2

[22] Guptasarma P, Balasubramanian D, Matsugo S, Saito I (1992) Hydroxyl radical mediated damage to proteins, with special reference to the crystallins. Biochemistry 31: 4296–4303. 1567875 10.1021/bi00132a021

[23] Finley EL, Dillon J, Crouch RK, Schey KL (1998) Identification of tryptophan oxidation products in bovine alpha-crystallin. Protein Sci 7: 2391–2397. 9828005 10.1002/pro.5560071116 PMC2143850

[24] Krieger-Liszkay A, Fufezan C, Trebst A (2008) Singlet oxygen production in photosystem II and related protection mechanism. Photosynth Res 98: 551–564. 18780159 10.1007/s11120-008-9349-3

[25] Pospíšil P (2009) Production of reactive oxygen species by photosystem II. Biochim Biophys Acta 1787: 1151–1160. 19463778 10.1016/j.bbabio.2009.05.005

[26] Hillier W, Wydrzynski T (1993) Increases in peroxide formation by the photosystem II oxygen-evolving reactions upon removal of the extrinsic 16, 22, and 33 kDa proteins are reversed by CaCl_2_ addition. Photosynth Res 38: 417–423. 24317998 10.1007/BF00046769

[27] Henmi T, Miyao M, Yamamoto Y (2004) Release and reactive-oxygen-mediated damage of the oxygen-evolving complex subunits of PSII during photoinhibition. Plant Cell Physiol 45: 243–250. 14988496 10.1093/pcp/pch027

[28] Virgin I, Styring S, Andersson B (1988) Photosystem II disorganization and manganese release after photoinhibition of isolated spinach thylakoid membranes. FEBS Lett 233: 408–412.

[29] Yamamoto Y, Akasada T (1995) Degradation of antenna chlorophyll-binding protein CP43 during photoinhibition of photosystem II. Biochemistry 34: 9038–9045. 7619803 10.1021/bi00028a012

[30] Yamamoto Y, Doi M, Tamura N, Nishimura M (1981) Release of polypeptides from highly active O_2_-evolving photosystem II preparations by tris treatment. FEBS Lett 133: 265–268.

[31] McKinnon NK, Reeves DC, Akabas MH (2011) 5-HT3 receptor ion size selectivity is a property of the transmembrane channel, not the cytoplasmic vestibule portals. J Gen Physiol 138: 453–466. 21948949 10.1085/jgp.201110686 PMC3182448

[32] Rochu D, Viguié N, Renault F, Crouzier D, Froment M-T, et al (2004) Contribution of the active-site metal cation to the catalytic activity and to the conformational stability of phosphotriesterase: temperature-and pH-dependence. Biochem J 380: 627–633. 15018612 10.1042/BJ20031861 PMC1224221

[33] Boda D, Nonner W, Valiskó M, Henderson D, Eisenberg B, et al (2007) Steric selectivity in Na channels arising from protein polarization and mobile side chains. Biophys J 93: 1960–1980. 17526571 10.1529/biophysj.107.105478 PMC1959557

[34] Waggoner CM, Pecoraro V, Yocum CF (1989) Monovalent cations (Na^+^, K^+^, Cs^+^) inhibit calcium activation of photosynthetic oxygen evolution. FEBS Lett 244: 237–240.

[35] Johnson PE, Creagh AL, Brun E, Joe K, Tomme P, et al (1998) Calcium binding by the N-terminal cellulose-binding domain from *Cellulomonas fimi* β-1,4-glucanase CenC. Biochemistry 37: 12772–12781. 9737854 10.1021/bi980978x

[36] Mehler AH, Knox WE (1950) The conversion of tryptophan to kynurenine in the liver II. The enzymatic hydrolysis of formylkynurenine. J Biol Chem 187: 431–438. 14794728

[37] Barry BA (2011) Proton coupled electron transfer and redox active tyrosines in photosystem II. J Photochem Photobiol B-Biol 104: 60–71. 10.1016/j.jphotobiol.2011.01.026 PMC3164834 21419640

[38] Macdonald GM, Steenhuis JJ, Barry BA (1995) A difference fourier-transform infrared spectroscopic study of chlorophyll oxidation in hydroxylamine-treated photosystem II. J Biol Chem 270: 8420–8428. 7721736 10.1074/jbc.270.15.8420

[39] Roose JL, Wegener KM, Pakrasi HB (2007) The extrinsic proteins of photosystem II. Photosynth Res 92: 369–387. 17200881 10.1007/s11120-006-9117-1

[40] Grossman AR, Bhaya D, Apt KE, Kehoe DM (1995) Light-harvesting complexes in oxygenic photosynthesis: diversity, control, and evolution. Annu Rev Genet 29: 231–288. 8825475 10.1146/annurev.ge.29.120195.001311

[41] Berlett BS, Stadtman ER (1997) Protein oxidation in aging, disease, and oxidative stress. J Biol Chem 272: 20313–20316. 9252331 10.1074/jbc.272.33.20313

[42] Hunzinger C, Wozny W, Schwall GP, Poznanović S, Stegmann W, et al (2006) Comparative profiling of the mammalian mitochondrial proteome: multiple aconitase-2 isoforms including *N*-formylkynurenine modifications as part of a protein biomarker signature for reactive oxidative species. J Proteome Res 5: 625–633. 16512678 10.1021/pr050377+

[43] Møller IM, Kristensen BK (2006) Protein oxidation in plant mitochondria detected as oxidized tryptophan. Free Radic Biol Med 40: 430–435. 16443157 10.1016/j.freeradbiomed.2005.08.036

[44] Krieger A, Rutherford AW, Vass I, Hideg E (1998) Relationship between activity, D1 loss, and Mn binding in photoinhibition of photosystem II. Biochemistry 37: 16262–16269. 9819218 10.1021/bi981243v

[45] Adir N, Zer H, Shochat S, Ohad I (2003) Photoinhibition-a historical perspective. Photosynth Res 76: 343–370. 16228592 10.1023/A:1024969518145

[46] Mizusawa N, Tomo T, Satoh K, Miyao M (2003) Degradation of the D1 protein of photosystem II under illumination *in vivo*: two different pathways involving cleavage or intermolecular cross-linking. Biochemistry 42: 10034–10044. 12924952 10.1021/bi0300534

[47] Mori H, Yamashita Y, Akasaka T, Yamamoto Y (1995) Further characterization of the loss of antenna chlorophyll-binding protein CP43 from photosystem II during donor-side photoinhibition. Biochim Biophys Acta 1228: 37–42.

[48] Rozak PR, Seiser RM, Wacholtz WF, Wise RR (2002) Rapid, reversible alterations in spinach thylakoid appression upon changes in light intensity. Plant Cell Environ 25: 421–429.

[49] Anderson JM, Aro E-M (1994) Grana stackig and protection of photosystem II in thylakoid membranes of higher plant leaves under sustained high irradiance: an hypothesis. Photosynth Res 41: 315–326. 24310114 10.1007/BF00019409

[50] Pätsikkä E, Kairavuo M, Šeršen F, Aro E-M, Tyystjärvi E (2002) Excess copper predisposes photosystem II to photoinhibition in vivo by outcompeting iron and causing decrease in leaf chlorophyll. Plant Physiol 129: 1359–1367. 12114589 10.1104/pp.004788 PMC166529

[51] Müller P, Li X-P, Niyogi KK (2001) Non-photochemical quenching. A response to excess light energy. Plant Physiol 125: 1558–1566. 11299337 10.1104/pp.125.4.1558 PMC1539381

[52] Hind G, Nakatani HY, Izawa S (1974) Light-dependent redistribution of ions in suspensions of chloroplast thylakoid membranes. Proc Natl Acad Sci U S A 71: 1484–1488. 4524652 10.1073/pnas.71.4.1484 PMC388254

[53] Ishijima S, Uchibori A, Takagi H, Maki R, Ohnishi M (2003) Light-induced increase in free Mg^2+^ concentration in spinach chloroplasts: measurement of free Mg^2+^ by using a fluorescent probe and necessity of stromal alkalinization. Arch Biochem Biophys 412: 126–132. 12646275 10.1016/s0003-9861(03)00038-9

[54] Barry BA (1995) Tyrosyl radicals in photosystem II. Methods Enzymol 258: 303–319. 8524157 10.1016/0076-6879(95)58053-0

[55] Anderson LB, Maderia M, Ouellette AJA, Putnam-Evans C, Higgins L, et al (2002) Posttranslational modifications in the CP43 subunit of photosystem II. Proc Natl Acad Sci U S A 99: 14676–14681. 12417747 10.1073/pnas.232591599 PMC137478

[56] Nixon PJ, Barker M, Boehm M, de Vries R, Komenda J (2005) FtsH-mediated repair of the photosystem II complex in response to light stress. J Exp Bot 56: 357–363. 15545296 10.1093/jxb/eri021

[57] Huesgen PF, Schuhmann H, Adamska I (2006) Photodamaged D1 protein is degraded in *Arabidopsis* mutants lacking the Deg2 protease. FEBS Lett 580: 6929–6932. 17157840 10.1016/j.febslet.2006.11.058

[58] Herman C, Prakash S, Lu CZ, Matouschek A, Gross CA (2003) Lack of a robust unfoldase activity confers a unique level of substrate specificity to the universal AAA protease FtsH. Mol Cell 11: 659–669. 12667449 10.1016/s1097-2765(03)00068-6

[59] Shacter E (2000) Quantification and significance of protein oxidation in biological samples. Drug Metab Rev 32: 307–326. 11139131 10.1081/dmr-100102336

[60] Berthold DA, Babcock GT, Yocum CF (1981) A highly resolved, oxygen-evolving photosystem II preparation from spinach thylakoid membranes. FEBS Lett 134: 231–234.

[61] Anderson LB, Ouellette AJA, Barry BA (2000) Probing the structure of photosystem II with amines and phenylhydrazine. J Biol Chem 275: 4920–4927. 10671529 10.1074/jbc.275.7.4920

[62] Lichtenthaler HK (1987) Chlorophylls and carotenoids-pigments of photosynthetic biomembranes. Methods Enzymol 148: 350–382.

[63] Ghanotakis DF, Babcock GT, Yocum CF (1984) Calcium reconstitutes high rates of oxygen evolution in polypeptide depleted photosystem II preparations. FEBS Lett 167: 127–130.

[64] Simat T, Meyer K, Steinhart H (1994) Synthesis and analysis of oxidation of carbonyl condensation compounds of tryptophan. J Chromatogr A 661: 93–99.

[65] Piccioni R, Bellemare G, Chua N (1982) In: Edelman H, Hallick RB, Chua N-H, editors. Methods in Chloroplast Molecular Biology. Amsterdam: Elsevier. 985–1014.

[66] Bollag DM, Edelstein SJ (1991) Protein Methods. New York: Wiley-Liss. 114–116 p.

[67] Ouellette AJA, Anderson LB, Barry BA (1998) Amine binding and oxidation at the catalytic site for photosynthetic water oxidation. Proc Natl Acad Sci U S A 95: 2204–2209. 9482863 10.1073/pnas.95.5.2204 PMC19295

[68] Towbin H, Staehelin T, Gordon J (1979) Electrophoretic transfer of proteins from polyacrylamide gels to nitrocellulose sheets: procedure and some applications. Proc Natl Acad Sci U S A 76: 4350–4354. 388439 10.1073/pnas.76.9.4350 PMC411572

