# Supplementary figures and images for: Light-Induced Oxidative Stress, N-Formylkynurenine, and Oxygenic Photosynthesis
Source: PLoS One. 2012 Jul 31;7(7):e42220. doi: 10.1371/journal.pone.0042220 (PMC3409137; doi:10.1371/journal.pone.0042220)

**Figure S1**


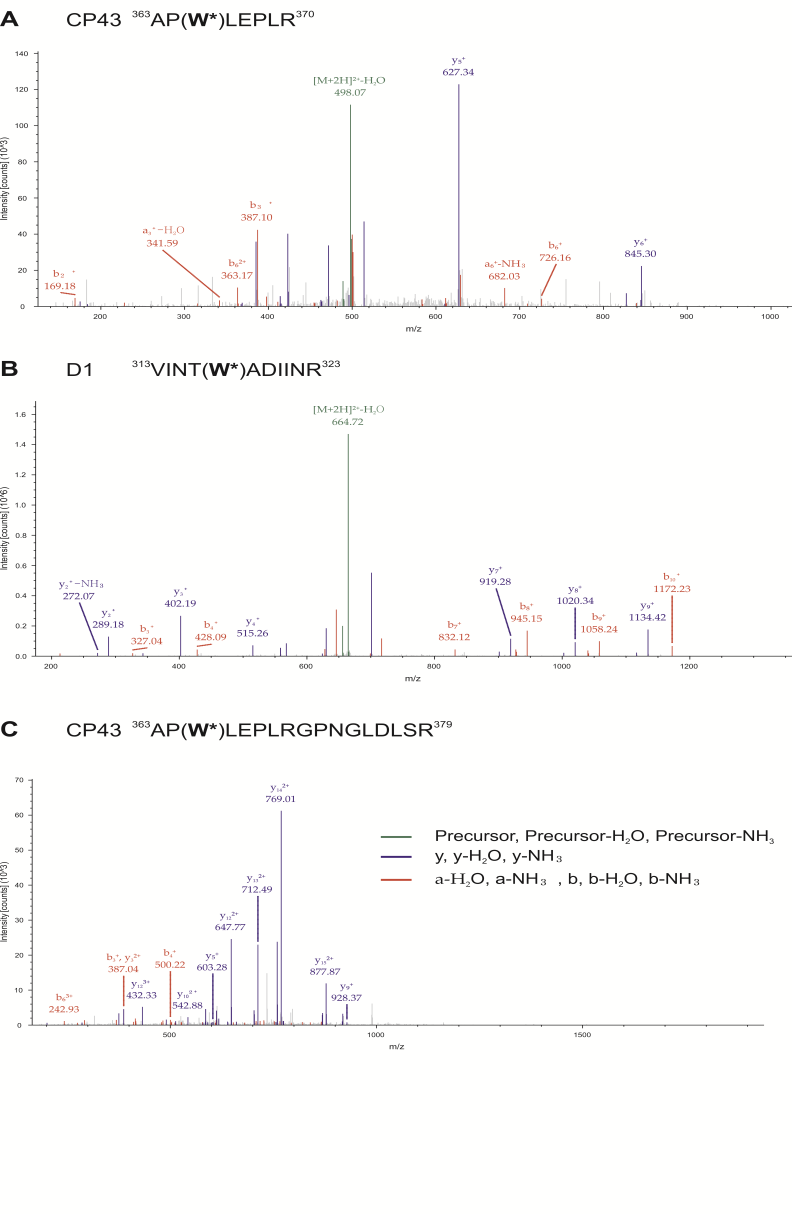

Supplement: Figure S1 — Representative MS/MS spectra of NFK modifications in CP43 (A and C) and D1 (B) proteins. The peaks in blue represent the b-fragments. The peaks in red represent the y-fragments. The NFK modified W is indicated in the corresponding sequences. This residue carries the +32 m/z masss shift, which was unambiguously assigned to Trp-365 in CP43 (A and C) and Trp-317 in D1 (B). (DOCX) [file pone.0042220.s001.docx]
