# Supplementary material for: Light-Induced Oxidative Stress, N-Formylkynurenine, and Oxygenic Photosynthesis
Source: PLoS One. 2012 Jul 31;7(7):e42220. doi: 10.1371/journal.pone.0042220 (PMC3409137; doi:10.1371/journal.pone.0042220)
Supplement: Table S1 — #NOD, not observed in dark; NODL, not observed in dark or light. (DOCX) [file pone.0042220.s002.docx]

**Table S1. Average light-induced changes in NFK yield (fold change) and average retention times (ret. time) of HPLC fractions A-D^#^**

| **Fraction** | **TM** | | **PSII** | | **PSII**  **+ Na_2_EDTA** | | **PSII**  **+ ZnCl_2_** | | **PSII**  **+ NaCl** | | **PSII**  **+ TMA** | | **TW PSII** | |
| --- | --- | --- | --- | --- | --- | --- | --- | --- | --- | --- | --- | --- | --- | --- |
|  | fold  change | ret.  time | fold  change | ret.  time | fold change | ret.  time | ret.  time | ret.  time | fold  change | ret.  time | fold  change | ret.  time | fold  change | ret.  time |
| A | 0.8 ± 0.6 | 25.6 | 0.9 ± 0.2 | 25.7 | 1.0 ± 0.2 | 25.3 | 1.0 ± 0.1 | 25.3 | 1.1 ± 0.2 | 25.7 | 0.9 ± 0.1 | 25.3 | nodl | nodl |
| B | nodl | nodl | nodl | nodl | nod | 26.1 | nod | 26.1 | nod | 26.1 | nod | 26.3 | nodl | nodl |
| C | 0.7 ± 0.2 | 27.3 | 2.1 ± 0.5 | 27.4 | 0.5 ± 0.2 | 27.6 | 1.1 ± 0.2 | 27.6 | 0.6 ± 0.2 | 27.6 | 0.8 ± 0.2 | 27.2 | 1.2 ± 0.3 | 27.4 |
| D | nodl | nodl | 2.4 ± 1.3 | 34.3 | 0.6 ± 0.3 | 34.4 | 1.1 ± 0.3 | 34.4 | 0.8 ± 0.5 | 34.3 | 0.9 ± 0.3 | 33.8 | 1.4 ± 0.2 | 34.3 |
