# Supplementary material for: Light-Induced Oxidative Stress, N-Formylkynurenine, and Oxygenic Photosynthesis
Source: PLoS One. 2012 Jul 31;7(7):e42220. doi: 10.1371/journal.pone.0042220 (PMC3409137; doi:10.1371/journal.pone.0042220)
Supplement: Table S2 — For the identification of peptides, filter criteria were set to warrant a false discovery rate of less than 1% on the peptide level. In each of the three independent LC-MS/MS runs of the four fractions, more than 20000 MS/MS spectra were recorded. For fraction A–C, between 3500–5000 spectra were assigned to peptides from 50–80 proteins from S. oleracea. In fraction D only, 1200 spectra could be assigned to peptides of about 50 proteins. (DOCX) [file pone.0042220.s003.docx]

**Table S2. Oxidative tryptophan modifications identified by LC-MS-MS in HPLC fractions A-D**

| Fraction | PSII subunit | Sequence | W  modification | M modification | MH^+^ (Da) | X-Corr |
| --- | --- | --- | --- | --- | --- | --- |
| A | CP43 | ^363^AP**W**LEPLR^370^ | NFK  (+32 *m/z*) |  | 1013.5407 | 2.80 |
|  |  |  | Kyn  (+4 *m/z*) |  | 985.5459 | 2.64 |
|  |  |  | OH-Trp  (+16 *m/z*) |  | 997.5453 | 2.64 |
|  | CP43 | ^363^AP**W**LEPLRGPNGLDLSR^379^ | Kyn  (+4 *m/z*) |  | 1895.0107 | 2.50 |
|  |  |  | OH-Trp  (+16 *m/z*) |  | 1907.0107 | 2.83 |
|  | CP43 | ^358^F**W**DLR^362^ | OH-Trp  (+16 *m/z*) |  | 752.3726 | 1.81 |
| B | D1 | ^313^VINT**W**ADIINR^323^ | NFK  (+32 *m/z*) |  | 1346.7029 | 3.46 |
|  |  |  | Kyn  (+4 *m/z*) |  | 1318.7095 | 4.10 |
|  |  |  | OH- Trp  (+16 *m/z*) |  | 1330.7095 | 4.01 |
| C | CP43 | ^363^AP**W**LEPLRGPNGLDLSR^379^ | NFK  (+32 *m/z*) |  | 1923.0086 | 2.01 |
|  |  |  | Kyn  (+4 *m/z*) |  | 1895.0108 | 1.94 |
|  |  |  | OH- Trp  (16 *m/z*) |  | 1907.0115 | 3.18 |
|  | CP43 | ^383^DIQPWQER^390^ | OH-Trp  (+16 *m/z*) |  | 1087.5131 | 1.54 |
|  | CP43 | ^358^F**W**DLR^362^ | OH-Trp  (+16 *m/z*) |  | 752.3715 | 1.75 |
| D | D2 | ^8^FTKDEKDLFDS**M**DD**W**LR^24^ | NFK  (+32 *m/z*) |  | 2192.9786 | 5.30 |
|  |  |  | Kyn  (+4 *m/z*) |  | 2164.9852 | 4.24 |
|  |  |  | OH-Trp  (16 *m/z*) |  | 2176.9814 | 5.74 |
|  |  |  | OH- Kyn  (+20 *m/z*) |  | 2180.9875 | 4.50 |
|  |  |  |  | Met-sulfoxide (+16 *m/z*) | 2176.9814 | 5.93 |
|  |  |  | NFK  (+32 *m/z*) | Met-sulfoxide (+16 *m/z*) | 2208.9739 | 5.53 |
|  |  |  | Kyn  (+4 *m/z*) | Met-sulfoxide (+16 *m/z*) | 2180.9767 | 5.61 |
|  |  |  | OH-Trp  (+16 *m/z*) | Met-sulfoxide (+16 *m/z*) | 2192.9789 | 5.38 |
| D | D2 | ^14^DLFDS**M**DD**W**LR^24^ | NFK  (+32 *m/z*) |  | 1444.6027 | 2.18 |
|  |  |  | OH-Trp  (16 *m/z*) |  | 1428.6091 | 3.07 |
|  |  |  | OH- Kyn  (+20 *m/z*) |  | 1432.6038 | 2.39 |
|  |  |  |  | Met-sulfoxide (+16 *m/z*) | 1428.6100 | 3.21 |
|  |  |  | NFK  (+32 *m/z*) | Met-sulfoxide (+16 *m/z*) | 1460.6058 | 2.49 |
|  |  |  | Kyn  (+4 *m/z*) | Met-sulfoxide (+16 *m/z*) | 1432.6038 | 3.03 |
|  |  |  | OH-Trp  (+16 *m/z*) | Met-sulfoxide (+16 *m/z*) | 1444.6027 | 2.45 |
